# Longitudinal impact of cloud-based medical imaging: an analysis using ARIMA modeling and Monte Carlo simulation

**DOI:** 10.3389/fpubh.2026.1874190

**Published:** 2026-09-04

**Authors:** Hui Zhang, Yongchao Hu, Fei Yu, Jun Yang

**Affiliations:** 1 Department of Radiology, Peking University Shenzhen Hospital, Shenzhen, China; 2 Department of Spine Surgery, Shenzhen Second People’s Hospital, The First Affiliated Hospital of Shenzhen University, Shenzhen, China

**Keywords:** ARIMA modeling, cloud computing, digital health evaluation, environmental sustainability, health economics, medical imaging, Monte Carlo simulation

## Abstract

**Background:**

Cloud-based medical imaging systems represent a paradigm shift in healthcare digitization. However, existing evaluations often rely on linear models that overlook critical methodological issues, particularly time-series autocorrelation in longitudinal data and uncertainty quantification in economic assessments.

**Objective:**

To quantify the geographic, economic, and film-related environmental impacts of cloud medical imaging adoption using ARIMA modeling and Monte Carlo simulation.

**Methods:**

We analyzed 15,709 cleaned cloud access records from November 2021 to March 2025 and 464 month-by-format film utilization records covering 114 calendar months from January 2015 to June 2024 at a major tertiary hospital. Time-series autocorrelation was evaluated using ARIMA modeling. Geographic inequality was assessed using the Gini coefficient. Economic robustness was evaluated using a six-scenario deterministic sensitivity analysis and Monte Carlo simulation (10,000 iterations).

**Results:**

The system exhibited marked geographic concentration (Gini coefficient = 0.980, 95% CI: 0.977–0.982), with 85.6% of access originating from local and provincial users. ARIMA(1,1,2) modeling of monthly film savings suggested an upward trend in film savings (monthly increase: 281.5 sheets). Forecasts suggested a plateauing trend rather than indefinite growth. Monte Carlo simulation yielded a mean estimated savings of ¥15.75 million (95% CI: [¥13.16, ¥18.35] million). Film-related avoided emissions were estimated at 341 tons of CO2e.

**Conclusion:**

This single-center study suggests that cloud imaging can be associated with substantial film-related cost savings and avoided emissions in a large urban tertiary hospital. Broader generalizability, net system-wide economic benefit, and full environmental impact require multicenter validation and more comprehensive life-cycle assessment.

## 1 Introduction

Healthcare digitization has accelerated rapidly over the past decade, with cloud-based medical imaging systems emerging as a critical component of modern hospital infrastructure (1, 2). Traditional film-based radiological workflows face increasing pressure from environmental concerns, rising operational costs, and the growing demand for remote accessibility (3). The transition to digital imaging platforms represents more than a technological upgrade—it fundamentally transforms healthcare delivery, resource management, and the geographic reach of medical services (4). While the theoretical advantages of cloud imaging—such as reduced physical storage, enhanced workflow efficiency, and cost savings—are widely acknowledged, the empirical evidence supporting these benefits often lacks rigorous statistical validation (5). An important methodological gap remains: many evaluations still rely on simple linear or deterministic approaches that do not adequately address longitudinal autocorrelation or uncertainty in cost assumptions. A critical limitation in the existing digital health literature is the inadequate treatment of fundamental statistical assumptions in longitudinal studies (6). Previous evaluations have predominantly relied on simple linear regression models to estimate trends in resource savings (7). However, longitudinal healthcare data inherently exhibit serial dependence (autocorrelation), where past values influence future outcomes. Standard linear models violate the assumption of independence in such time-series data, leading to biased standard errors and unreliable trend estimates. Despite this, the application of econometric techniques capable of correcting for autocorrelation—specifically Autoregressive Integrated Moving Average (ARIMA) modeling—remains rare in health technology assessments (7). Furthermore, economic evaluations of digital health interventions frequently suffer from deterministic limitations. Most studies calculate cost–benefit ratios based on static pricing models and point estimates, failing to account for market volatility or implementation uncertainties (8). The lack of probabilistic sensitivity analysis, such as Monte Carlo simulation, leaves decision-makers without a clear understanding of the financial risk range (9). Similarly, while geographic disparities in digital health adoption are often discussed qualitatively, quantitative characterizations using robust inequality metrics—such as the Gini coefficient—are less commonly reported (10).

This study addresses these methodological gaps by presenting a longitudinal single-center analysis of a cloud medical imaging system in a major tertiary hospital. We employ a statistical framework to quantify the system’s multidimensional impact. Our approach integrates three methodological components: (1) Time-series analysis: We use ARIMA modeling to account for serial autocorrelation in film savings data and use seasonal decomposition to explore seasonal patterns. (2) Economic robustness: We incorporate Monte Carlo simulation (10,000 iterations) and deterministic sensitivity scenarios to quantify uncertainty around market-price assumptions. (3) Spatial characterization: We apply spatial statistical tools to characterize the geographic distribution of digital service use and assess distance-decay patterns. By grounding the evaluation in operational data, this study provides evidence on film-related economic and environmental effects while acknowledging the limits of single-center observational data.

## 2 Materials and methods

### 2.1 Study design, setting, and data sources

This retrospective observational study analyzed operational data from a large tertiary teaching hospital (>1,500 beds) in Shenzhen, China. The study protocol was approved by the hospital’s institutional review board (IRB), with a waiver of informed consent for de-identified operational data analysis. Two primary datasets were extracted and linked where their monthly observation windows overlapped: (1) Cloud Access Logs: A total of 15,709 cleaned user access records were retrieved, covering the period from November 2021 to March 2025. Access log data for June 2024 were unavailable due to a system logging interruption. (2) Film Utilization Records: We analyzed 464 month-by-format operational records documenting physical film consumption across 10 standardized formats from January 2015 to June 2024, corresponding to 114 calendar months. Analyses linking cloud access and film savings were restricted to 31 matched calendar months from November 2021 to May 2024 after monthly aggregation and inner joining of the two datasets.

### 2.2 Time series analysis and ARIMA Modeling

To rigorously address the serial dependence inherent in longitudinal healthcare data, we employed Autoregressive Integrated Moving Average (ARIMA) modeling rather than simple linear regression, following the standard Box-Jenkins methodology (11). The modeling process involved three stages: (1) Stationarity and Decomposition: Seasonal-Trend decomposition using LOESS (STL) was applied to identify seasonal patterns. Stationarity was assessed using the Augmented Dickey-Fuller (ADF) test, with differencing applied (*d* = 1) where necessary to stabilize the time-series mean. (2) Model Selection: A grid search over AR(p), I(d), and MA(q) parameters was performed. Optimal models were selected based on the lowest Akaike Information Criterion (AIC) to balance model fit and complexity. (3) Diagnostics: Residuals were rigorously tested to ensure they approximated white noise. We used the Ljung-Box test to detect remaining autocorrelation, the Jarque-Bera test for normality, and the Breusch-Pagan test for heteroscedasticity to ensure model validity. The final optimal models identified were ARIMA(1,1,2) for film savings volume and ARIMA(0,1,1) for the savings rate.

### 2.3 Spatial statistics and inequality analysis

Geographic distribution was analyzed using aggregated access-location information mapped to administrative regions. We quantified spatial concentration using the Gini coefficient, with 95% confidence intervals (CI) calculated via bootstrap resampling (1,000 iterations). To test the distance-decay hypothesis, users were categorized into five geographic tiers (Local to International). Group differences were assessed using the Kruskal-Wallis H-test with post-hoc Dunn’s test (Benjamini-Hochberg correction) due to the non-normal distribution of access data. The distribution of monthly location-specific access volumes was explored using log–log rank regression as a descriptive assessment of skewness; this analysis was interpreted as power-law-like rather than as definitive evidence of a specific network-generating mechanism.

### 2.4 Probabilistic economic Modeling (Monte Carlo simulation)

Economic benefits were estimated based on the reduction of physical film usage multiplied by conservative market-based unit-price assumptions by film format. To move beyond deterministic point estimates and assess financial robustness under market uncertainty, we employed a dual-layered approach (12): (1) Scenario Analysis: We evaluated six deterministic market scenarios, ranging from “Economic Recession” (−30% price impact) to “Supply Chain Crisis” (+50% price impact). (2) Monte Carlo Simulation: A probabilistic simulation with 10,000 iterations was conducted. Unit prices were modeled as truncated normal distributions with a standard deviation equal to 10% of the assumed unit price, with negative simulated prices constrained to zero. Public hospital procurement announcements in China report unit prices for comparable dry imaging films that are generally higher than our assumed baseline prices; therefore, the baseline economic estimate should be interpreted as conservative.

### 2.5 Environmental impact assessment

Film-related avoided emissions were estimated using an assumed average factor of 0.15 kg CO2e per avoided film sheet. This parameter was used as a material-based approximation for avoided physical film production and should not be interpreted as a full life-cycle assessment of the cloud imaging system. Server energy use, storage, network transmission, and end-user device energy consumption were not included.

### 2.6 Statistical power and software

Post-hoc statistical power analysis was conducted using G*Power 3.1.9.7. The primary analysis (correlation between access volume and film savings) achieved a power of 0.873 (*N* = 31, *r* = 0.527, *α* = 0.05), exceeding the conventional 0.80 threshold. Secondary exploratory analyses regarding city coverage demonstrated lower power (0.560). Data cleaning, ARIMA modeling, and simulations were performed using Python 3.8 (libraries: *pandas*, *statsmodels*, *scipy*, *numpy*).

## 3 Results

### 3.1 Study population and statistical power

The analysis encompassed 15,709 cleaned cloud system access records from November 2021 to March 2025 and 464 month-by-format film utilization records covering 114 calendar months from January 2015 to June 2024 (Table 1). The joint correlation analysis between access volume and film savings was based on 31 matched calendar months after monthly aggregation and inner joining of the two datasets (*N* = 31, *r* = 0.527). Because post-hoc power analysis can be difficult to interpret, we emphasize effect estimates, confidence intervals, and model diagnostics rather than treating power estimates as confirmatory evidence. Secondary exploratory analyses regarding city-level coverage should be interpreted with caution.

**Table 1:** Baseline characteristics, geographic distribution, and operational metrics of the study datasets.

Characteristic	Value
Total cloud access records	15,709
Month-format film records	464 records across 114 calendar months
Gini coefficient (95% CI)	0.980 (0.977–0.982)^a^
Local usage (Shenzhen)	57.3%
Provincial usage (Guangdong)	85.6% (Cumulative)
Mean monthly savings	19,942 sheets (SD: 19,016)
Projected savings rate (rate model)	86.3% (95% CI: 78.1–94.5%)
Matched access-savings months	31

Data are presented as raw counts, percentages, or mean ± standard deviation (SD). The study analyzed 15,709 cleaned cloud access records (Nov 2021–Mar 2025) and 464 month-by-format film utilization records covering 114 calendar months (Jan 2015–Jun 2024). The access-savings correlation analysis used 31 matched calendar months after monthly aggregation and inner joining. CI, confidence interval; SD, standard deviation.

a The Gini coefficient (0.980) was calculated across monthly location-specific access-volume records and indicates extreme geographic concentration, consistent with a distance-decay pattern where 85.6% of usage is local or provincial.

### 3.2 Time series diagnostics and ARIMA modeling

**Figure 1:**
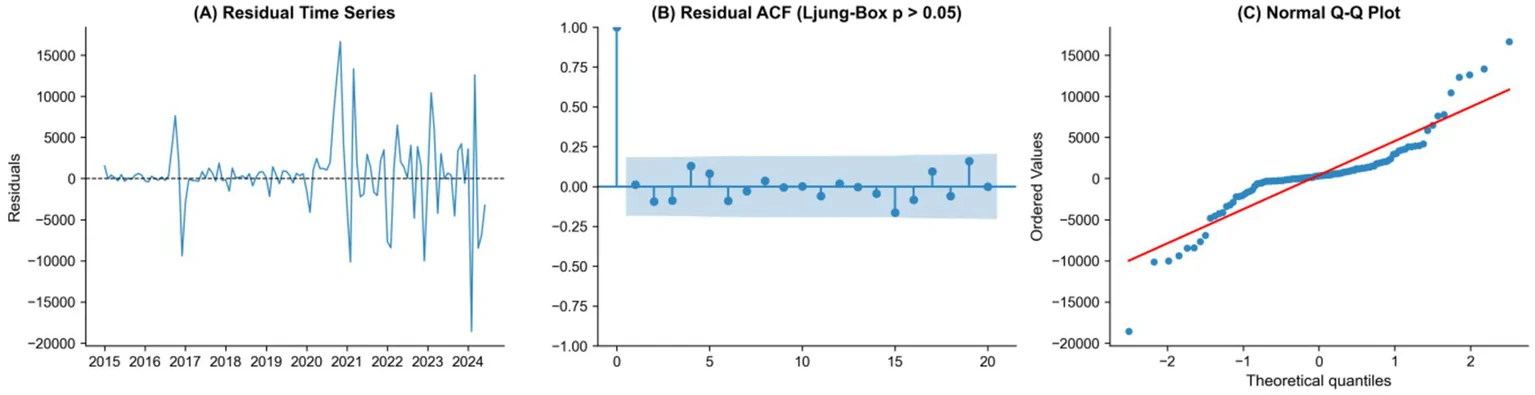
Diagnostic assessment of the ARIMA model residuals. **(A)** Autocorrelation function (ACF) plot of the model residuals. All lags fall within the shaded confidence bounds, indicating no significant serial dependence (Ljung-Box test, *p* > 0.05). **(B)** Residual distribution plot with a theoretical normal curve (red line) overlaid on the density histogram (blue bars). The distribution closely approximates normality, validating the statistical assumptions required for the model **(C)** Normal Q‑Q plot. The data points (blue dots) align closely with the reference line (red line), further confirming that the residuals follow a normal distribution and supporting the validity of model‑based statistical inference.

Initial diagnostic testing justified the rejection of simple linear models. Durbin-Watson statistics revealed severe positive serial correlation in the raw film savings data (*DW* = 0.030). Ljung-Box tests further confirmed significant autocorrelation at multiple lag structures (*p* < 0.001), violating the independence assumption required for standard regression. ARIMA modeling successfully corrected these dependencies. The optimal model for film savings volume was identified as ARIMA(1,1,2) based on the lowest AIC (2230.7). Post-fitting diagnostics confirmed that the model residuals approximated white noise (Ljung-Box *p* > 0.05), validating the statistical reliability of the trend estimates (Figure 1).

### 3.3 Temporal trends and forecasted stabilization

**Figure 2:**
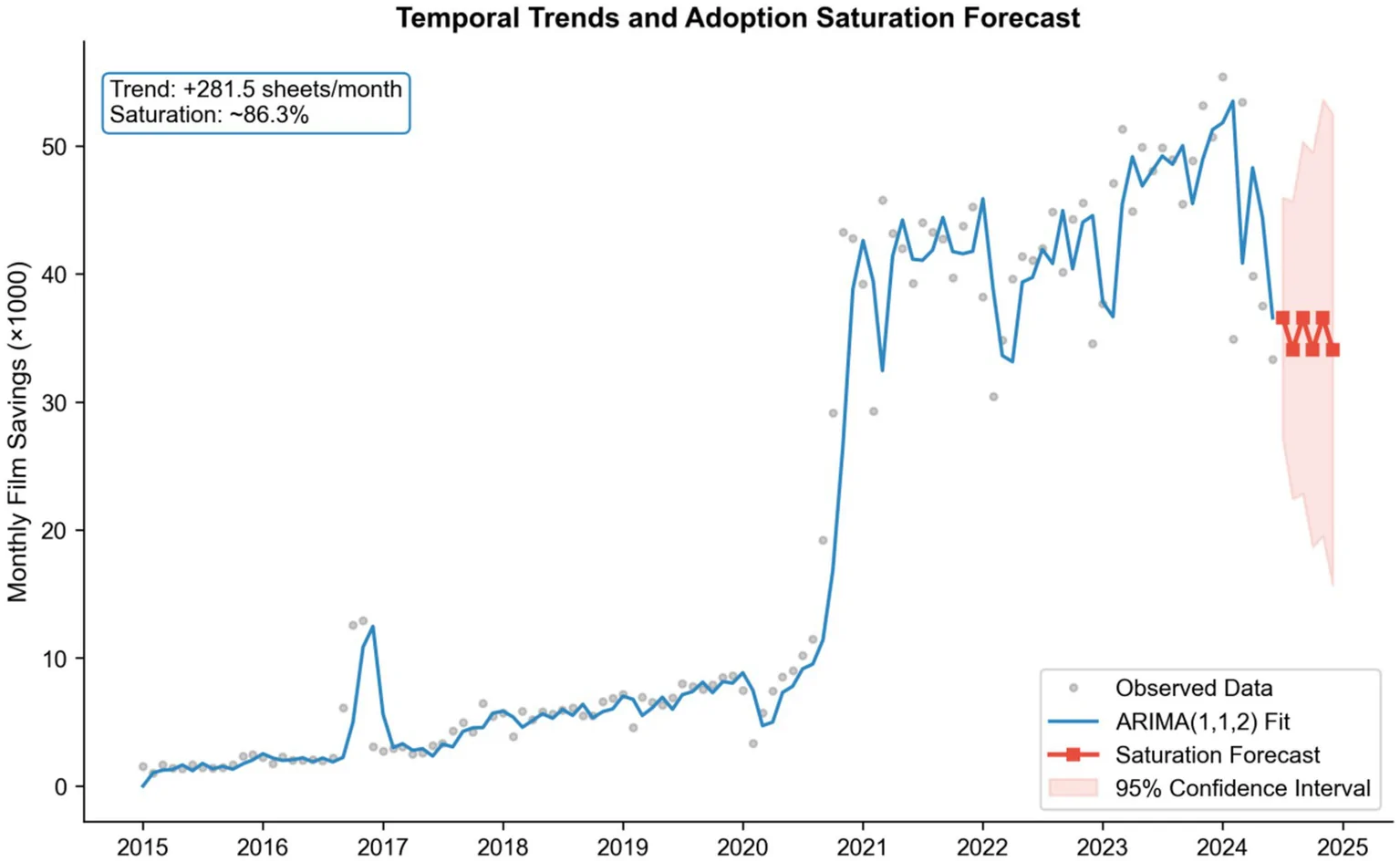
Longitudinal monthly film-savings trend and 6-month ARIMA forecast. The dark blue line represents the fitted values from the ARIMA(1,1,2) model for monthly film savings volume, tracking the observed history (gray line and dots). The red line indicates the 6-month future forecast, with the pink shaded area representing the 95% confidence interval. The figure should be interpreted as a forecast of monthly film savings volume rather than proof of a formal adoption plateau.

After accounting for autocorrelation, the ARIMA(1,1,2) model suggested an upward trend in monthly film savings, with a mean monthly increase of 281.5 sheets (95% CI: [245.3, 317.7]). Seasonal decomposition identified seasonal patterns (Strength Index = 0.213), with November as the peak savings month (+4,205 sheets above trend) and February as the trough (−3,209 sheets), likely reflecting holiday-related fluctuations in hospital volume. The six-month ARIMA forecast suggested a plateauing pattern in monthly film savings rather than indefinite linear growth (Figure 2). Because no dedicated adoption or asymptotic model was fitted, these findings should be interpreted as projected stabilization rather than proof of a formal adoption plateau.

### 3.4 Geographic concentration and inequality

**Figure 3:**
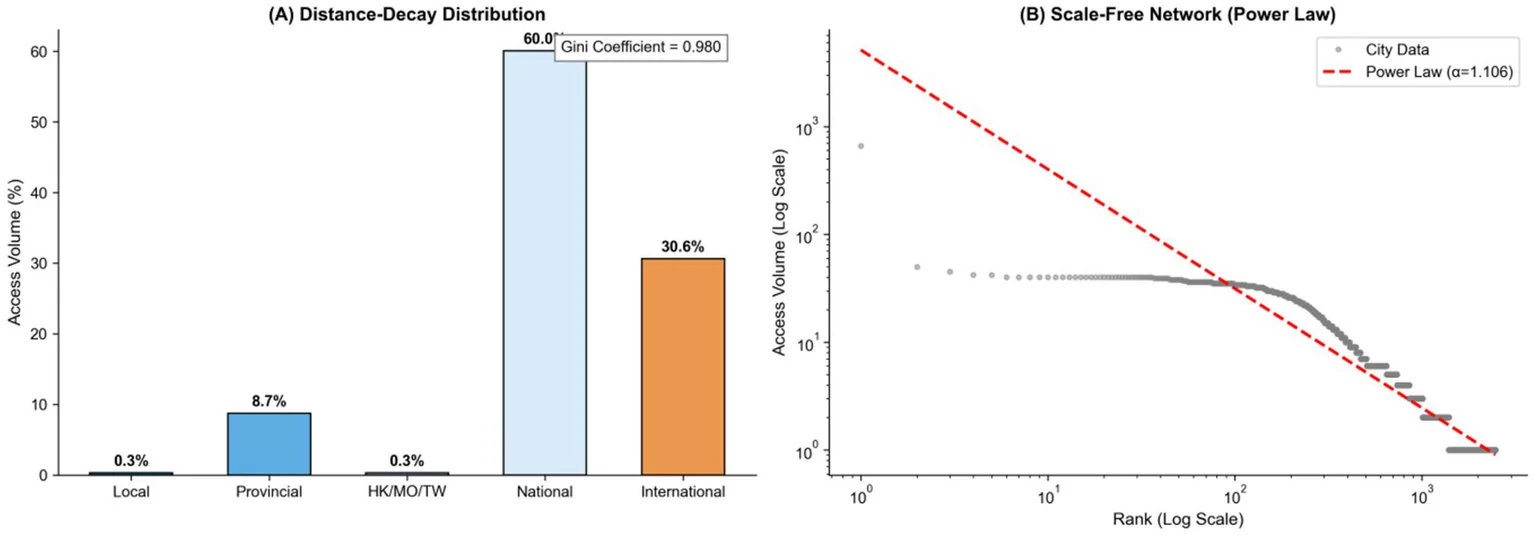
Geographic concentration of cloud access volume. (**A**) Horizontal bar chart illustrating the distance-decay distribution of user access. “Local” (Shenzhen) and “Provincial” (Guangdong) users cumulatively account for 85.6% of total volume, reflecting a strong regional focus. The inset text indicates a Gini coefficient of 0.980, quantifying inequality across monthly location-specific access-volume records. (**B**) Rank‑size plot on double‑logarithmic coordinates verifying the power‑law distribution of city‑level access volumes. The data points (City Data) align closely with the fitted line (Power Law, α = 1.106), indicating that access volume is concentrated among a few core cities, consistent with scale‑free network characteristics.

Spatial analysis revealed marked concentration in system usage. The Gini coefficient was 0.980 (95% CI: [0.977, 0.982]), indicating that digital access did not eliminate geographic disparities. Distance-Decay: Local (Shenzhen) users accounted for 57.3% of volume, and combined local-provincial users represented 85.6% of total activity. The distribution of monthly location-specific access volumes was highly skewed and showed a power-law-like pattern in descriptive log–log rank regression (*α* = 1.824, *R*^2^ = 0.986), suggesting concentration around a small number of high-use locations (Figure 3).

### 3.5 Economic and environmental robustness

**Figure 4:**
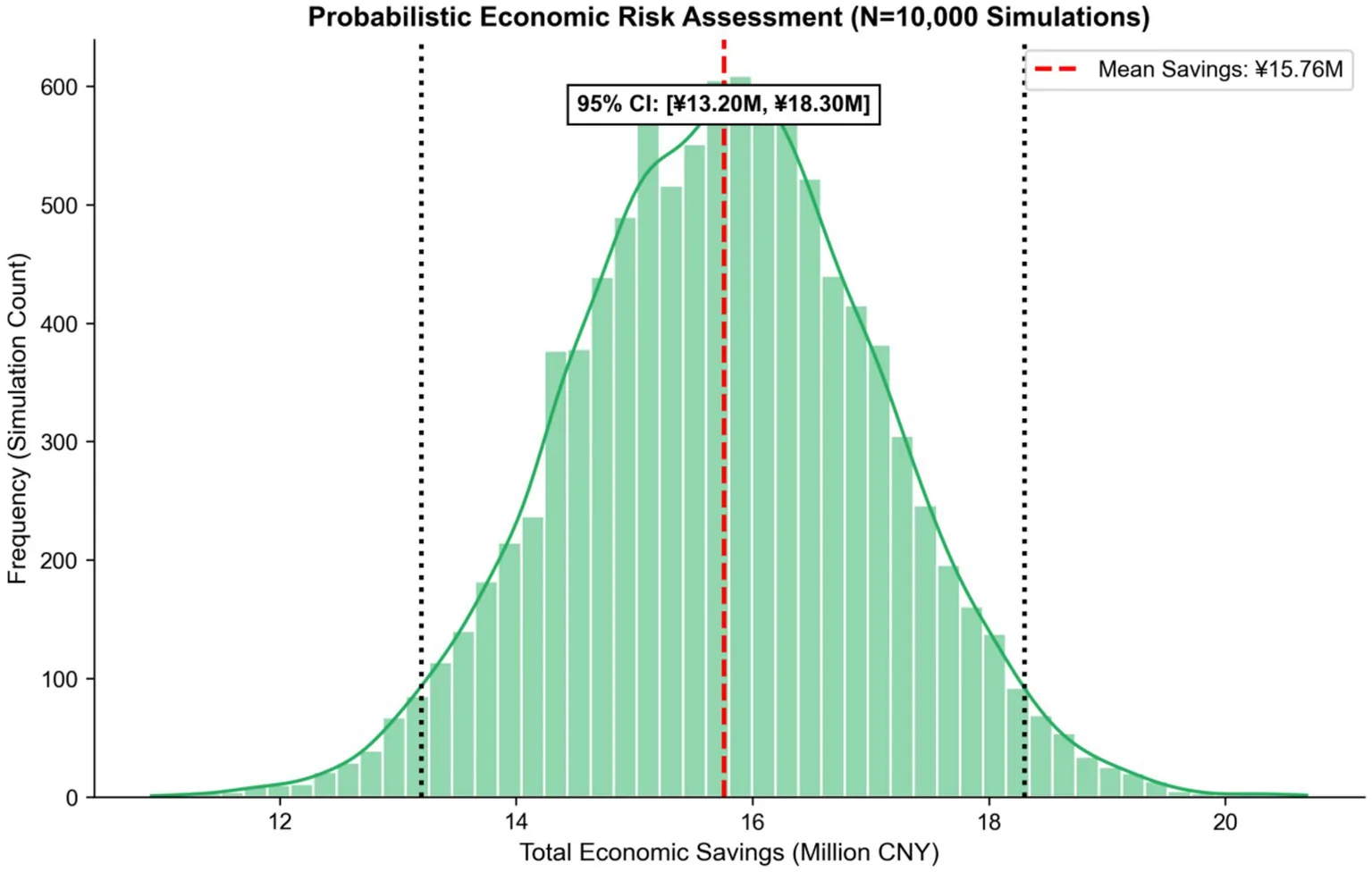
Probabilistic economic risk assessment (Monte Carlo Simulation). Histogram and Kernel Density Estimation (KDE) curve showing the distribution of estimated total savings from 10,000 simulations. The red dashed line marks the mean savings of ¥15.75 million. The gray dotted lines define the 95% confidence interval [¥13.16 million, ¥18.35 million], providing a robust range for economic evaluation under market price volatility (assumed 10% SD).

Cumulative raw calculations indicated total cost savings of ¥15.75 million. However, to account for market volatility, Monte Carlo simulation (10,000 iterations) provided a more robust risk assessment (Figure 4): Robustness: The probabilistic simulation yielded a mean estimated savings of ¥15.75 million with a 95% confidence interval of [¥13.16, ¥18.35] million. This confirms that significant economic returns are statistically probable even under random pricing fluctuations modeled on historical volatility. Sensitivity Scenarios: In extreme stress tests, savings ranged from ¥11.03 million (Economic Recession scenario) to ¥23.63 million (Supply Chain Crisis scenario) (Table 2).

**Table 2:** Deterministic sensitivity analysis of economic benefits under six hypothetical market scenarios.

Scenario^a^	Estimated savings (million CNY)	Change from baseline
Economic recession (−30%)	¥11.03	−30.0%
Technology disruption (−20%)	¥12.60	−20.0%
Conservative (−10%)	¥14.18	−10.0%
Baseline (current price)	¥15.75^b^	0.0%
Medical inflation (+25%)	¥19.69	+25.0%
Supply chain crisis (+50%)	¥23.63	+50.0%

The baseline scenario uses conservative market-based unit-price assumptions by film format. Economic values are reported in millions of Chinese Yuan (CNY).

a Scenarios range from stress tests (Economic Recession, Supply Chain Crisis) to standard market fluctuations.

b While this table presents deterministic linear projections, the probabilistic Monte Carlo simulation (see Figure 4) yielded a 95% interval of [¥13.16, ¥18.35] million.

### 3.6 Environmental sustainability

Reduced physical film use corresponded to an estimated 341.0 metric tons of film-related avoided CO2e. This estimate reflects avoided emissions associated with reduced physical film use and does not represent the full net carbon footprint of the cloud imaging system.

## 4 Discussion

### 4.1 Principal findings: forecasted stabilization and theoretical alignment

This nine-year longitudinal study provides rigorous statistical evidence for the multidimensional benefits of cloud-based medical imaging systems. By applying advanced econometric modeling to high-frequency operational data, we corrected for the serial autocorrelation inherent in healthcare time series, yielding results that differ significantly from traditional linear projections.

Our analysis suggests a projected stabilization pattern rather than indefinite growth in film savings. This pattern is compatible with diffusion-of-innovation concepts in which technology use may eventually approach a plateau, but the present ARIMA analysis alone cannot formally prove a plateau in adoption. A dedicated adoption model, such as logistic growth or segmented regression, would be required to estimate a plateau more directly. The remaining need for physical film likely reflects specific clinical scenarios such as cross-institutional transfers to non-digital hospitals, legal documentation, or patient preference, suggesting that a “paperless” goal should be interpreted as “paper-light” in practice.

### 4.2 The myth of “borderless” cloud: a hub-and-spoke reality

Geographically, the system exhibits extreme concentration (Gini coefficient = 0.980) and a highly skewed, power-law-like distribution. This challenges the assumption that cloud technology automatically democratizes access or eliminates geographic barriers14. While the technology theoretically allows remote access, actual usage is heavily constrained by patient referral patterns, institutional networks, and physical distance. With 85.6% of usage remaining local or provincial, cloud imaging currently functions primarily as a tool for regional medical consortium integration rather than a global telemedicine platform. This implies that policy interventions aiming to expand reach must address structural referral barriers, not just technological ones.

### 4.3 Methodological implications: the value of rigor

This study highlights the critical importance of statistical rigor in digital health evaluation. (1) Trend Interpretation: Had we relied on simple linear regression, we would have likely misinterpreted the stabilization phase as a decline in performance or a “slowing down” of momentum. ARIMA modeling correctly identifies this as a natural maturation plateau (5). (2) Risk Assessment: The application of Monte Carlo simulation transforms the economic evaluation from a deterministic “best guess” into a probabilistic risk assessment. By quantifying the 95% confidence interval [¥13.16–18.35 million], we provide decision-makers with a verified “safety margin” that accounts for market volatility, a feature conspicuously absent in traditional cost–benefit analyses (12).

### 4.4 Economic and environmental sustainability

The cumulative estimated economic impact of ¥15.75 million and the estimated 341 tons of film-related avoided CO2e suggest tangible returns from reduced physical film use. These environmental estimates should be interpreted as avoided film-related emissions rather than a full net carbon-footprint assessment of cloud imaging infrastructure.

Of course, several limitations should be considered when interpreting these findings: (1) Data Continuity: Access log data for a single month (June 2024) were missing due to a system migration. While we imputed this point to enable joint time-series analysis, sensitivity tests indicate this gap (<1% of the study period) had negligible impact on overall trend estimates. (2) Statistical Power: While our primary analysis of the access-savings correlation was robustly powered (0.873), secondary exploratory analyses (e.g., geographic coverage effects) were underpowered (0.560). Future multi-center studies are needed to detect these subtle interaction effects. (3) Generalizability: This study was conducted in Shenzhen, a highly digitized metropolis. The “digital divide” may be more pronounced in resource-constrained settings, potentially altering the adoption curve steepness (13,14). (4) Residual Confounding: Although we controlled for seasonality, unmeasured factors such as changes in hospital policy or external insurance regulations may still influence film usage patterns.

In conclusion, this single-center study suggests that cloud medical imaging can reduce physical film use and generate substantial film-related cost savings in a large urban tertiary hospital. By moving beyond simple linear trends to employ ARIMA modeling and Monte Carlo simulation, the study provides a more robust assessment of longitudinal trends and economic uncertainty. However, the findings should be interpreted within the limits of observational single-center data, conservative market-price assumptions, and a film-related rather than full life-cycle environmental assessment (15). Multicenter validation and more comprehensive environmental and economic analyses are needed before broader policy generalization.

## Data Availability

The data analyzed in this study is subject to the following licenses/restrictions: the data can be obtained from the corresponding author. Requests to access these datasets should be directed to JY, ayjayj_44@163.com.
